# *APOE* ε4, white matter hyperintensities, and cognition in Alzheimer and Lewy body dementia

**DOI:** 10.1212/WNL.0000000000008377

**Published:** 2019-11-05

**Authors:** Saira Saeed Mirza, Usman Saeed, Jo Knight, Joel Ramirez, Donald T. Stuss, Julia Keith, Sean M. Nestor, Di Yu, Walter Swardfager, Ekaterina Rogaeva, Peter St. George Hyslop, Sandra E. Black, Mario Masellis, Alzheimer's Disease Neuroimaging Initiative

**Affiliations:** From the Division of Neurology, Department of Medicine (S.S.M., D.T.S., S.E.B., M.M.), Hurvitz Brain Sciences Research Program (S.S.M., J.R., D.T.S., D.Y., W.S., S.E.B., M.M.) and LC Campbell Cognitive Neurology Research Unit (U.S., J.R., S.M.N., D.Y., W.S., S.E.B., M.M.), Sunnybrook Research Institute, Institute of Medical Science (U.S., S.E.B., M.M.), Rehabilitation Sciences Institute (D.T.S., S.E.B.), and Department of Psychiatry (S.M.N.), Faculty of Medicine, Heart and Stroke Foundation Canadian Partnership for Stroke Recovery (J.R., D.Y., W.S., S.E.B.) and Department of Anatomic Pathology (J.K.), Sunnybrook Health Sciences Centre, Department of Psychology, Faculty of Arts and Science (D.T.S.), Department of Pharmacology & Toxicity (D.Y., W.S.), Tanz Centre for Research in Neurodegenerative Diseases (E.R., P.S.G.H.), and Institute of Biomaterials and Biomedical Engineering (S.E.B.), University of Toronto, Canada; Data Science Institute and Medical School (J.K.), Lancaster University, Lancaster; and Cambridge Institute for Medical Research (P.S.G.H.), Department of Clinical Neuroscience, University of Cambridge, UK.

## Abstract

**Objective:**

To determine if *APOE* ε4 influences the association between white matter hyperintensities (WMH) and cognitive impairment in Alzheimer disease (AD) and dementia with Lewy bodies (DLB).

**Methods:**

A total of 289 patients (AD = 239; DLB = 50) underwent volumetric MRI, neuropsychological testing, and *APOE* ε4 genotyping. Total WMH volumes were quantified. Neuropsychological test scores were included in a confirmatory factor analysis to identify cognitive domains encompassing attention/executive functions, learning/memory, and language, and factor scores for each domain were calculated per participant. After testing interactions between WMH and *APOE* ε4 in the full sample, we tested associations of WMH with factor scores using linear regression models in *APOE* ε4 carriers (n = 167) and noncarriers (n = 122). We hypothesized that greater WMH volume would relate to worse cognition more strongly in *APOE* ε4 carriers. Findings were replicated in 198 patients with AD from the Alzheimer's Disease Neuroimaging Initiative (ADNI-I), and estimates from both samples were meta-analyzed.

**Results:**

A significant interaction was observed between WMH and *APOE* ε4 for language, but not for memory or executive functions. Separate analyses in *APOE* ε4 carriers and noncarriers showed that greater WMH volume was associated with worse attention/executive functions, learning/memory, and language in *APOE* ε4 carriers only. In ADNI-I, greater WMH burden was associated with worse attention/executive functions and language in *APOE* ε4 carriers only. No significant associations were observed in noncarriers. Meta-analyses showed that greater WMH volume was associated with worse performance on all cognitive domains in *APOE* ε4 carriers only.

**Conclusion:**

*APOE* ε4 may influence the association between WMH and cognitive performance in AD and DLB.


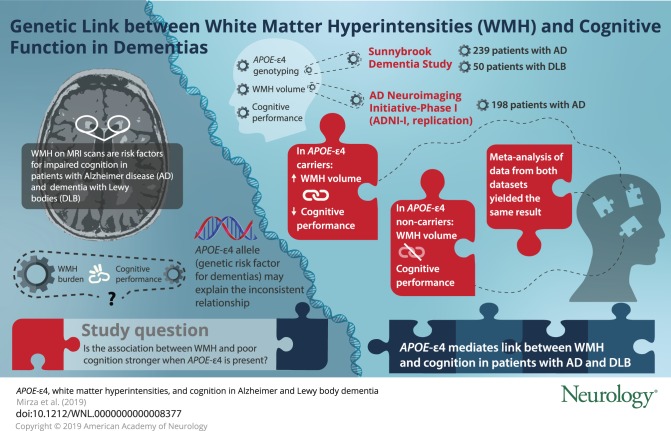


White matter hyperintensities (WMH) observed on structural MRI indicate cerebral small vessel disease (SVD) in most cases,^1^ are risk factors for cognitive impairment and Alzheimer disease (AD),^2,3^ and are prevalent in dementia with Lewy bodies (DLB).^4,5^ However, observed cognitive performance clinically does not always reflect the severity of the WMH burden.^6,7^

There are several reasons for the complex association between WMH and cognition: the etiology of WMH is heterogeneous, including vascular compromise and ischemia, venous collagenosis, leading to vasogenic edema,^8,9^ cerebral amyloid angiopathy (CAA), or a combination of these,^10^ and genetic vulnerability to neurodegeneration.

The *APOE* ε4 allele is the strongest known genetic risk factor for sporadic AD, and is a risk factor for DLB,^11,12^ CAA,^13^ and SVD.^14^ Despite these associations, it remains unknown if *APOE* ε4 modulates the relationship between WMH and cognition across the dementias, i.e., if *APOE* ε4 is an effect modifier in this association.

Therefore, we examined the role of *APOE* ε4 on the association between WMH and cognitive domains in patients with AD and patients with DLB with varying degrees of SVD. We tested associations with domain-specific cognitive impairment instead of global cognition because at different disease stages, impairment might be more apparent in certain domains and not others. We hypothesized that (1) higher WMH burden would be more strongly associated with worse cognition in *APOE* ε4 carriers than noncarriers and the association would be *APOE* ε4 allele dosage dependent, (2) this association would be irrespective of the clinical diagnosis, and (3) if indeed WMH burden is associated with worse cognition in *APOE* ε4 carriers, WMH in carriers might be a result of a more toxic vascular pathology, i.e., CAA.

## Methods

This is a cross-sectional study examining the effect of *APOE* ε4 on the association of WMH volume and cognitive functions in patients with AD and patients with DLB.

### Setting

This work was embedded within the Sunnybrook Dementia Study (SDS), a prospective observational study of patients with dementia.^15^ The majority of participants in the SDS are Caucasian of European descent.

For replication of study findings, data from the Alzheimer's Disease Neuroimaging Initiative Phase I (ADNI-I) (2004) were utilized.^16^ ADNI was launched in 2003 as a public–private partnership. For the most up to date information, see adni-info.org.

ADNI-I is characterized by a low WMH burden (<10 cm^3^) at recruitment and cognitive impairment is largely attributed to AD pathology with minimal confounding comorbid SVD. The SDS represents a heterogeneous real-world clinical case series followed longitudinally, and reflects a similar vascular risk factor and SVD burden profile to community and population-based studies.^17^

### Standard protocol approvals, registrations, and patient consents

SDS (ClinicalTrials.gov: NCT01800214) is approved by the local Research Ethics Board at Sunnybrook Health Sciences Centre and written informed consent was obtained from participants or their surrogate caregivers according to the Declaration of Helsinki.

### Study samples

#### SDS sample

Data from 289 MRI-confirmed stroke-free patients with dementia, including *APOE* ε4 genotype, MRI volumetrics, and neuropsychological battery, were available. This included 239 patients with AD and 50 patients with DLB with varying degrees of SVD. Of the 289 patients included, 36 had autopsy data available.

#### ADNI-I (replication sample)

A total of 198 patients with AD with *APOE* ε4 genotype, MRI volumetric, and neuropsychological data available were included. We used data from the 24 month follow-up visit instead of baseline for better comparability to the SDS sample given the mild initial nature of participants included in ADNI, i.e., progression of the AD stage and that of WMH burden, and ensuring a sufficient number of participants to obtain valid estimates.

### Diagnosis of dementia

For both study samples, AD was diagnosed on recruitment, using the Neurologic and Communicative Disorders and Stroke and Alzheimer's Disease and Related Disorders Association criteria,^18^ while DLB (SDS only) was diagnosed using the Third Report of DLB Consortium criteria.^19^ Diagnoses were confirmed on clinical follow-up.

Diagnostic consensus in the SDS was achieved through review by at least 2 physicians (M.M. and S.E.B.) with expertise in dementia diagnosis.

### *APOE* ε4 genotyping

*APOE* genotyping was performed using DNA extracted from leukocytes in both SDS^20^ and ADNI.^21^ Genotype frequencies in both samples did not deviate from that predicted by Hardy-Weinberg equilibrium.

### MRI (WMH volume)

#### SDS sample

MRI scans were acquired on a 1.5T Signa system (GE Healthcare, Milwaukee, WI). Three sets of structural MRI sequences were used: T1-weighted, T2-weighted, and proton density–weighted (PD). Details of MRI acquisition are provided elsewhere.^15^

MRIs were processed using the Semi-Automated Brain Region Extraction and Lesion Explorer processing pipeline.^22^ WMHs were identified as lesions that appear as punctate or diffuse regions of hyperintense signal on T2/PD MRI. These images were used to quantify global, deep, and periventricular WMH volumes (cm^3^). For analyses, total WMH volumes adjusted for total intracranial volume (TIV) were used: TIV adjusted WMH volumes = (raw WMH volume/TIV) × 10^3^.

#### ADNI-I (replication sample)

Methods for MRI data acquisition, processing, and WMH quantification are described in detail elsewhere.^23^

### Neuropsychological test battery

#### SDS sample

The neuropsychological battery was performed within 90 days of MRI acquisition. Trained psychometrists blinded to neuroimaging, dementia diagnosis, and genotype information administered all tests.^24^ The following tests for global cognition and domain-specific functioning were administered: (1) Mini-Mental State Examination (MMSE); (2) Dementia Rating Scale; (3) California Verbal Learning Test (CVLT), total acquisition score through 5 trials, CVLT long delay–free recall, and CVLT long delay–cued recall; (4) Wechsler Memory Scale (WMS) visual recognition immediate and delayed recall; (5) forward digit span (FDS); (6) backward digit span (BDS); (7) Boston Naming Test (BNT); (8) Semantic Fluency (SF); (9) Wisconsin Card Sorting Test (WCST); (10) Controlled Oral Word Association task–Phonemic Fluency (PF-FAS); (11) Trail-Making Test A; and (12) Digit Symbol Substitution Task (DSST). The number of patients who completed each test differed; this variability was dependent on dementia severity. Ninety percent of patients had completed at least 8 neuropsychological tests.

#### ADNI-I (replication sample)

The cognitive test battery in ADNI-1 included (1) MMSE; (2) Rey Auditory Verbal Learning Test (RAVLT), total acquisition score through 5 trials and delayed recall; (3) logical memory immediate and delayed recall; (4) FDS; (5) BDS; (6) BNT; (7) category fluency (animals and vegetables); (8) Trail-Making Test A; and (9) DSST. Details are described elsewhere.^25^

For all test scores, higher scores correspond to better cognition, except for WCST (number of nonperseverative errors; SDS only), and Trail-Making Test A (time taken to complete the task in seconds), for which a higher score corresponds to worse performance.

### Covariates

#### SDS sample

Age, sex, years of education, diabetes mellitus type 2 (present vs absent), systolic and diastolic blood pressure (mm Hg), hypertension (present vs absent), smoking status (never, past, or current smoking), and dementia diagnosis (AD or DLB) were considered potential confounders.

#### ADNI-I (replication sample)

Available covariates in ADNI-I included age, sex, education, and systolic and diastolic blood pressure.

For consistency across both study samples, we included systolic and diastolic blood pressure as covariates and not hypertension.

### Neuropathology methods in SDS (exploratory sample)

Thirty-six of the SDS cases had a postmortem neuropathologic examination to diagnose and stage neurodegenerative disease phenomena.^15^ This workup included a screen for CAA using immunohistochemistry for β-amyloid (Aβ) (Dako, Glostrup, Denmark; Mach 4 detection system) in at least 2 brain sections (cerebellum and frontal cortex). For 34 of these 36 cases, the original autopsy reports were reviewed by a neuropathologist (J.K.) to determine the presence or absence of CAA. For 2 of the 36 cases, the reports were not available. For 3 of the 34 cases with available reports, the presence or absence of amyloid angiopathy was not stated in the autopsy report; the slides from the original autopsy were retrieved, reviewed by J.K., and the presence or absence of CAA was determined. Given that only 2 anatomical areas of the brain had been screened for CAA, applying a formal CAA grading scheme was not feasible. Using these data (n = 34), we aimed to explore if there was a higher prevalence of CAA in *APOE* ε4 carriers.

### Statistical analyses

TIV-adjusted WMH volumes were log-transformed to achieve a normal distribution and standardized by calculating *z* scores.

We compared participant characteristics between *APOE* ε4 carriers and noncarriers using *t* tests for continuous and χ^2^ tests for categorical variables.

#### Confirmatory factor analysis and regression

In both samples, we aimed to reduce the number of tests by making comprehensive factor scores (latent constructs) for each cognitive domain, based on the specific tests and the domain that they are known to assess. Therefore, we conducted a Confirmatory Factor Analysis (CFA)^26^ and calculated scores for each cognitive factor, i.e., attention/executive functions, learning/memory, and language for each participant. These cognitive factor scores were then used as outcomes in our analyses instead of individual test scores. CFA uses all available information for any model specified instead of a complete case analysis, and obtained factors are allowed to correlate. We present standardized measures in this article to facilitate interpretation. Adequacy of model fit to the data was assessed by comparative fit index (CFI) (range 0–1; recommended ≥0.95), root mean square error of approximation (RMSEA) (range 0–1; recommended ≤0.06), and the standardized root mean square residual (SRMR) (range 0–1; recommended ≤0.08).^27^

Subsequently, in both study samples, we first tested associations between WMH volume and each of the 3 cognitive factor scores with all covariates including *APOE* ε4 carrier status as a predictor, and also tested the interaction between WMH and *APOE* ε4 carrier status.

Second, we investigated the associations between WMH volume and each cognitive factor score in *APOE* ε4 carriers and noncarriers separately, based on our a priori hypothesis, i.e., higher WMH burden would be more strongly associated with worse cognition in *APOE* ε4 carriers than noncarriers, because of the known strong biological effects of the *APOE* ε4 allele.^28^

#### SDS sample

Relationships between the following cognitive factors and observed test scores were hypothesized and tested using CFA: (1) attention/executive functions (FDS, BDS, Trails A, WCST–perseverative errors, PF-FAS, DSST), (2) learning/memory (CVLT–total acquisition score, trials 1–5, CVLT–long delay free and cued recall, WMS–immediate recall, delayed recall), and (3) language (BNT, SF, PF-FAS). Scores for WCST and Trails A were inverse-coded for consistency with other test scores.

We used the following multiple linear regression model in the SDS sample (n = 289) to test associations of WMH with executive functions, memory, and language, and an interaction between WMH and *APOE* ε4 carrier status:

Cognitive factor score = β0 + β1 * WMH volume + β2 * *APOE* ε4 carrier status + β3 * (WMH volume × *APOE* ε4 carrier status) + β4 * age + β 5 * sex + β6 * education + β7 * diabetes mellitus + β8 * systolic blood pressure + β9 * diastolic blood pressure + β10 * smoking + β11 * clinical dementia diagnosis.

Further, we tested associations of WMH with the cognitive domains in *APOE* ε4 carriers and noncarriers separately using a similar model, but without *APOE* ε4 and its interaction term.

For each regression, 2 models were fitted. Model I was adjusted for age and sex; II was additionally adjusted for years of education, diabetes mellitus type 2, systolic and diastolic blood pressure, smoking status, and dementia diagnosis. We also repeated model II by replacing systolic and diastolic blood pressure by hypertension.

The following variables had missing values and were dealt with by multiple imputation using chained equations in Stata: systolic and diastolic blood pressure and smoking (2.8%, n = 8), diabetes (3.1%, n = 9), and years of education (0.3%, n = 1). All available covariates were used as predictors for imputation.

Since studies suggest that WMH are not associated with cognition in DLB, but in AD only,^4,5^ we repeated the analyses in *APOE* ɛ4 carriers and noncarriers excluding DLB cases.

In a post hoc analysis, we tested if associations between WMH and cognitive domains in *APOE* ɛ4 carriers were dependent on *APOE* ε4 allele dosage. After comparing study characteristics and WMH volumes by *APOE* ɛ4 allele dosage (0, 1, or 2 alleles) using analysis of variance (ANOVA) (Tukey post hoc) and χ^2^ tests for continuous and categorical variables, respectively, we repeated our analyses in *APOE* ɛ4 heterozygotes (n = 130) and *APOE* ɛ4 homozygotes (n = 37).

We explored the prevalence of CAA by *APOE* ɛ4 carrier status in our autopsy subsample (n = 34). This analysis was conditional on our primary results, i.e., to be performed if indeed WMH were associated with worse cognition more strongly in *APOE ε4* carriers than noncarriers. In this case, we hypothesized that since *APOE* ε4 is a risk factor for CAA, the likely etiology of WMH in carriers is CAA, which might be more toxic than WMH caused by vascular compromise or ischemia due to cardiovascular risk factors alone. We compared the numbers of patients with CAA by *APOE* ε4 carrier status and by allele dosage using the Fisher exact test. Since studies suggest that CAA is more prevalent in *APOE* ε2 carriers,^29^ we also examined the number of persons with CAA across genotypes: ε2-ε3 (n = 2), ε3-ε3 (n = 12), ε3-ε4 (n = 13), and ε4-ε4 (n = 7); however, statistical comparisons could not be made due to small numbers within some cells.

#### ADNI-I (replication sample)

Relationships between the following cognitive factors and observed test scores were hypothesized and tested: (1) attention/executive (FDS, BDS, Trail-Making Test A [inverse-coded], DSST), (2) learning/memory (RAVLT trials 1–5 [immediate recall], RAVLT delayed recall, logical memory immediate and delayed recall), and (3) language (BNT, category fluency–animals, category fluency–vegetables).

As in the SDS, a full model with an interaction term (WMH × *APOE* ε4) was tested (full ADNI-1 sample; n = 198), and then analyses were repeated in *APOE* ε4 carriers and noncarriers separately. For regression, model I was adjusted for age and sex only; II was additionally adjusted for education and systolic and diastolic blood pressure. Analyses were also repeated in *APOE* ε4 heterozygotes (n = 91) and homozygotes (n = 40).

Since power was limited in both our study samples, we meta-analyzed the β-coefficients from SDS and ADNI-I for all 3 cognitive scores to obtain more robust estimates.^30^ This was done using the *metan* command in Stata,^31^ which uses inverse variance weighting method.

Level of significance was set at 0.05 (two-sided) for all statistical tests, and all analyses were performed using Stata Software Version 14.1 (StataCorp, College Station, TX).

### Data availability

The authors have carefully documented all data, methods, and materials used to conduct the research in this article and agree to share anonymized data by request from any qualified investigator.

## Results

### SDS sample

Characteristics of the study sample are presented in table 1. Participant characteristics or WMH volumes did not differ between *APOE* ε4 carriers and noncarriers. Table 2 summarizes the neuropsychological test scores by *APOE* ε4 carrier status.

**Table 1:**
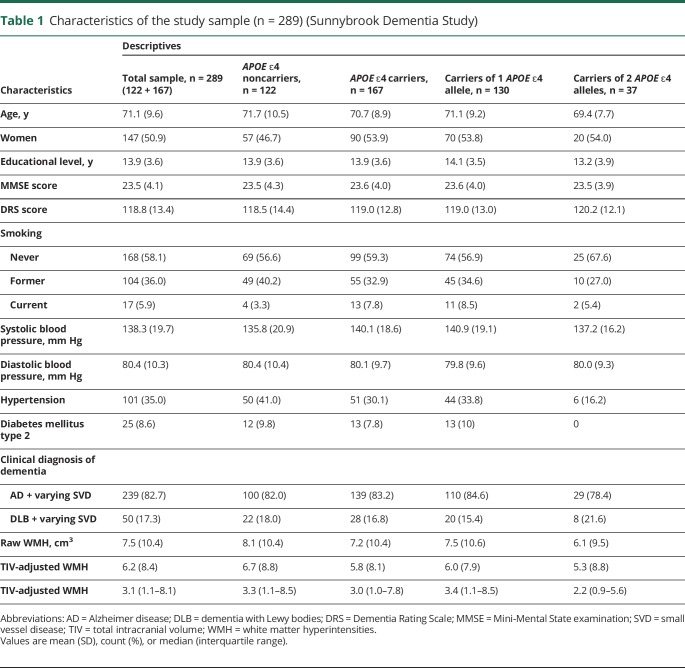
Characteristics of the study sample (n = 289) (Sunnybrook Dementia Study)

**Table 2:**
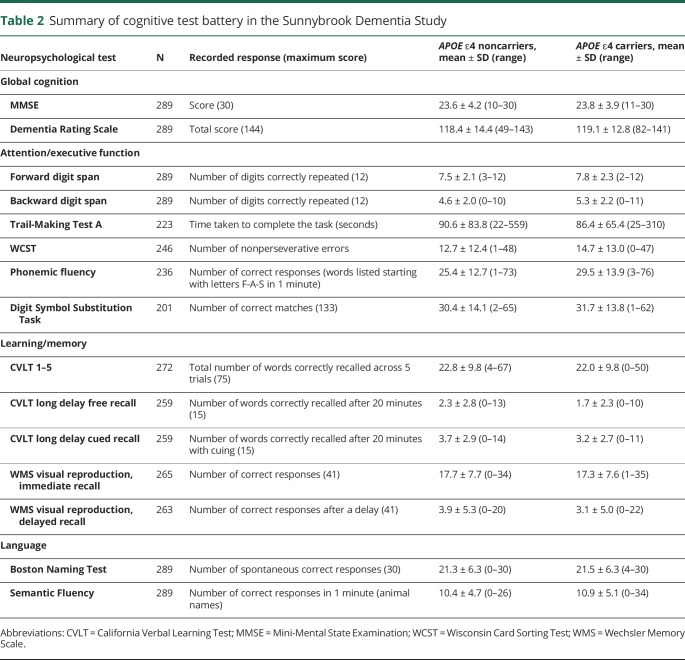
Summary of cognitive test battery in the Sunnybrook Dementia Study

In the CFA, single confirmatory factor models for all 3 cognitive factors tested showed excellent fit to the data: attention/executive (CFI 0.98, RMSEA 0.04, SRMR 0.03), learning/memory (CFI 0.99, RMSEA 0.04, SRMR 0.009), and language (CFI 1.00, RMSEA <0.0001, SRMR <0.0001).

In the full model (n = 289), WMH volume was not associated with attention/executive functions, learning/memory, or language. An interaction between WMH and *APOE* ε4 (*p* value 0.02) was observed for language, but not for executive functions (*p* value 0.26) or memory (*p* value 0.11). With our a priori hypothesis that WMH relate to cognition differently in carriers and noncarriers, and a significant interaction observed between WMH and *APOE* ε4 for language, we performed analyses separately in *APOE* ε4 carriers and noncarriers for all cognitive domains.

In these analyses, greater WMH volumes were associated with worse attention/executive functions, learning/memory, and language in only *APOE* ε4 carriers; no associations were observed in noncarriers (table 3). Replacing blood pressure with hypertension did not change results.

**Table 3:**
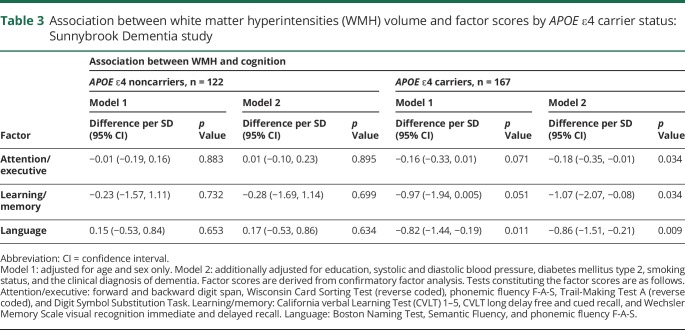
Association between white matter hyperintensities (WMH) volume and factor scores by *APOE* ε4 carrier status: Sunnybrook Dementia study

After excluding patients with DLB (n = 50), a similar pattern of results was obtained (table 4).

**Table 4:**
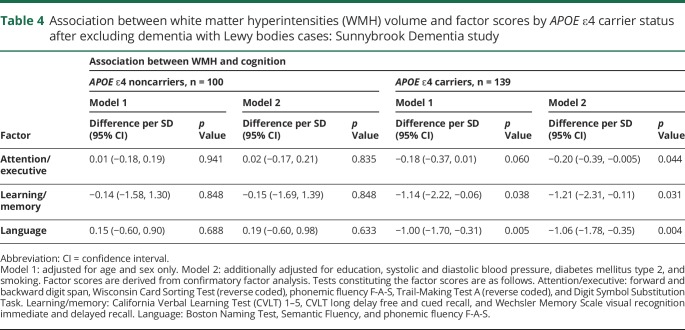
Association between white matter hyperintensities (WMH) volume and factor scores by *APOE* ε4 carrier status after excluding dementia with Lewy bodies cases: Sunnybrook Dementia study

Homozygous *APOE* ε4 carriers were younger than noncarriers and heterozygous carriers (ANOVA *p* value < 0.001). Homozygous *APOE* ε4 carriers also had lower WMH volume than noncarriers and heterozygous carriers (ANOVA *p* value = 0.002). Heterozygous carriers had a greater burden of cardiovascular risk factors (table 1).WMH were related to worse attention/executive functions (difference per SD −0.23; 95% confidence interval [CI] −0.41, −0.04), learning/memory (difference per SD −1.39; 95% CI −2.51, −0.26), and language (difference per SD −0.90; 95% CI −1.59, −0.22) in *APOE* ε4 heterozygotes only, and not in homozygotes (difference in attention/executive score per SD 0.06; 95% CI −0.37, 0.49; difference in learning/memory score per SD 0.21; 95% CI −2.21, 2.63; difference in language score per SD 0.34; 95% CI −2.14, 1.45).

### Exploratory neuropathology sample: SDS

In the autopsy subsample, 21 patients were neuropathologically diagnosed with AD and 15 with DLB. All AD cases were pathologically confirmed to have AD, including one case with coexisting Lewy bodies. All DLB cases were confirmed to have DLB, with varying degrees of neurofibrillary tangle pathology.^15^ A total of 66.6% (n = 8/12) of the *APOE* ε4 noncarriers had CAA compared to 76% (n = 16/21) of *APOE* ε4 carriers. Sixty-four percent (n = 9/14) of heterozygous *APOE* ε4 carriers had CAA, whereas 100% (n = 7/7) of the homozygous *APOE* ε4 carriers had CAA. However, differences across these groups were not significant (Fisher exact test *p* value = 0.123). Fifty percent (n = 6/12) of patients with ε3-ε3 genotype had CAA, 50% (n = 1/2) of the ε3-ε2 patients, 39% (n = 8/13) of ε3-ε4 patients, and 100% (n = 7/7) of the ε4-ε4 patients had CAA. There were no patients with ε2-ε2 genotype.

### ADNI-I (replication sample)

Characteristics of the study sample are summarized in table 5. We did not find any differences in characteristics and WMH volumes between *APOE* ε4 carriers and noncarriers except that carriers were significantly younger than noncarriers (*p* value 0.02).

**Table 5:**
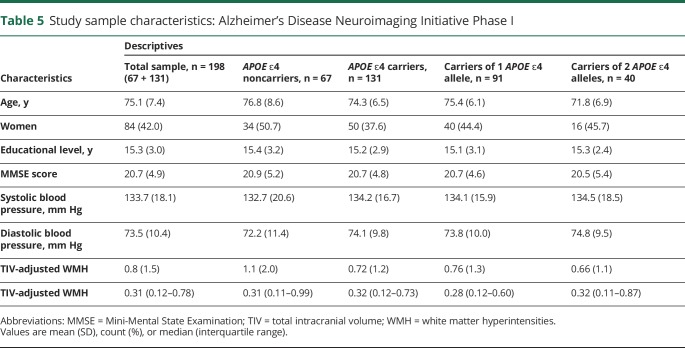
Study sample characteristics: Alzheimer's Disease Neuroimaging Initiative Phase I

Comparison of study characteristics by allele dosage showed that *APOE* ε4 homozygotes were younger than heterozygotes and noncarriers (ANOVA *p* value ≤ 0.001; table 5). WMH volumes did not differ by allele dosage. Table 6 summarizes the neuropsychological test scores by *APOE* ε4 carrier status for ADNI-I.

**Table 6:**
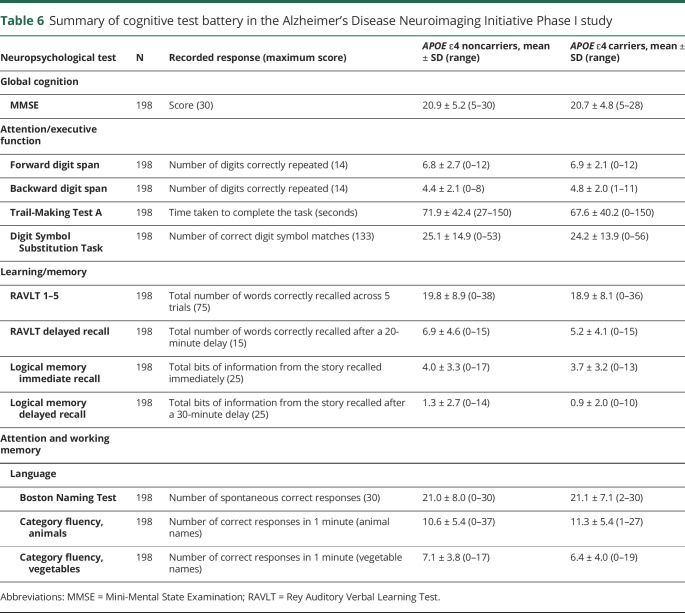
Summary of cognitive test battery in the Alzheimer's Disease Neuroimaging Initiative Phase I study

In the CFA, single confirmatory factor models for all 3 cognitive factors tested showed an excellent fit to the data: attention/executive (CFI 0.999, RMSEA ≤0.0001, SRMR 0.004), learning/memory (CFI 0.996, RMSEA 0.06, SRMR 0.019), and language (CFI 1.00, RMSEA ≤0.0001, SRMR <0.0001).

In the full model (n = 198), WMH volume was associated with attention/executive functions (*p* value <0.001), but not with memory or language. No interaction was observed between WMH and *APOE* ε4 for executive functions (*p* value 0.069), memory (0.97), or language (0.34).

In *APOE* ε4 carriers only, greater WMH volume was associated with worse performance on the attention/executive functions and language, but not with memory (table 7).

**Table 7:**
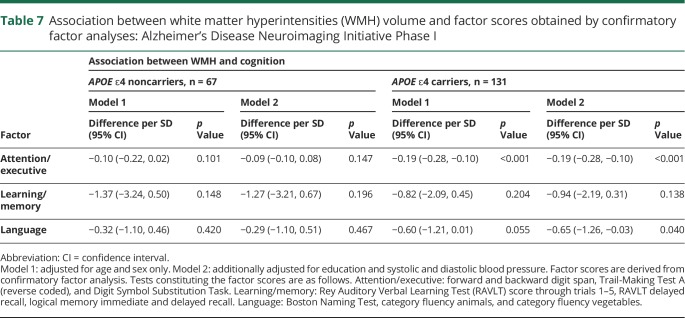
Association between white matter hyperintensities (WMH) volume and factor scores obtained by confirmatory factor analyses: Alzheimer's Disease Neuroimaging Initiative Phase I

As in the SDS, WMH volume was associated with executive functions in *APOE* ε4 heterozygotes (difference per SD −0.20; 95% CI −0.30, −0.09) but not in homozygotes (difference in score −0.23; 95% CI −0.47, 0.002). For language, however, effect estimates for both homozygotes and heterozygotes were nonsignificant.

Meta-analyses of estimates from SDS and ADNI-I showed a strong association of WMH with attention/executive functions (difference per SD −0.19; 95% CI −1.27, −0.11; *p* value 2.117 × 10^−3^), learning/memory (difference per SD −1.02; 95% CI −1.79, −0.25; *p* value 0.009), and language (difference per SD −0.75; 95% CI −1.19, −0.31; *p* value 0.0009) in carriers, with no effects seen in noncarriers. No heterogeneity was observed between the 2 studies and variance in effect estimates attributable to heterogeneity for all domains was ∼0%.

## Discussion

Our findings imply that in carriers of the *APOE* ε4 allele, WMH burden, a marker of cerebral SVD, is inversely associated with cognitive performance, whereas no such effect was seen in noncarriers. Moreover, this was consistent across the AD/DLB spectrum, in contrast to previous studies.^4,5^ After excluding patients with DLB from the SDS sample, the associations of WMH volume with executive functions, memory, and language remained significant. Cerebral SVD can be considered a relevant copathology across the AD/DLB spectrum. Because of the high frequency of coexisting neurodegenerative pathologies,^32,33^ shared risk factors and pathologies cannot be disentangled if samples are segregated on clinical diagnoses alone.^15^

Although a unified model with an interaction term is the optimum method to test effect modification, an important limitation is that more statistical power is required than for association testing, and thus false-negative results may be seen in smaller samples. The documented strong biological effects of *APOE* ε4^28^ formed the basis of our a priori hypothesis; that is, greater WMH burden relates more strongly with worse cognition in *APOE* ε4 carriers, which is why we also tested associations separately in carriers and noncarriers irrespective of the interaction results. Given the strong biological rationale, limited sample size, and a significant interaction observed for the language domain, this was a valid approach, which has also been used by other groups.^34,35^ However, studies in larger sample sizes are warranted.

The replication of worse executive functions and language in relation to higher WMH in ADNI-I *APOE* ε4 carriers is remarkable, and also validates our findings. Notably, ADNI-I comprises cases with relatively lower WMH burden compared to SDS,^17^ and this finding indicates that *APOE* ε4 may contribute to worse cognitive performance in those with even lower burden of cerebral SVD. Effect estimates for memory did not reach significance in the ADNI-I sample, which might be explained by lack of power. However, the significant association of greater WMH volume with cognitive impairment across all 3 domains observed in the meta-analysis supports our primary findings.

While our data supported our hypothesis, it failed to show an allele dosage effect. This could be a result of the small size of the homozygous group; however, the similar pattern of results in both SDS and ADNI-I suggests that this is not just a power issue. There are several possible considerations. The first consideration is age and cardiovascular risk factor distribution. Although in both study samples, age did not differ between *APOE* ε4 carriers and noncarriers, among carriers, homozygotes were younger. In the SDS sample, the homozygous group was not only younger, but it also had less WMH and cardiovascular risk factor burden, which might explain our findings. Second, since we adjusted for these pertinent confounders, a complex interaction may exist among *APOE* ε4, vascular risk factors, WMH, and cognition.^36,37^ Specifically, a higher vascular risk factor burden combined with *APOE* ε4 genotype results in reduced white matter integrity and predicts faster cognitive decline.^37^ Third, the observed association might also be dependent on the disease stage in addition to age, such that the association of WMH and cognition becomes more apparent with advancing age and dementia progression.^38^ Increasing age becomes an important determinant of cognitive decline when effects of *APOE* ε4 and its interactions with other risk factors are at play.^39,40^

The mechanisms underlying this association may be Aβ-dependent, Aβ-independent, or both. In addition to causing accelerated cerebral amyloid deposition and impaired clearance of Aβ, *APOE* ε4 can cause detrimental effects on brain through vascular pathways. *APOE* ε4 is associated with neurovascular dysfunction, has a synergistic effect with atherosclerosis by disrupting cholesterol homeostasis, and also affects vessels via CAA. These synergistic effects can drastically compound the damaging effects of WMH in *APOE* ε4 carriers.^41^ Faster WMH progression rates were noted in *APOE* ε4–positive patients with AD and healthy adults, supporting our interaction hypothesis.^38,42^ *APOE* ε4 carriers might also have more covert white matter damage that is not detected by routine imaging,^43^ but is reflected as worse cognitive outcomes. Future large prospective studies are needed.

WMH burden reflects a worse cerebrovascular status, potentially increasing vulnerability to neurodegeneration. Higher WMH volume has been associated with reduced cerebral perfusion both in hyperintense areas and normal-appearing white matter.^44^ Normal-appearing white matter surrounding WMH already exhibits subtle damage,^43^ and will likely develop into areas of T2 MRI-detectable WMH. Also, neuroinflammation is a key feature in AD,^45^ and *APOE* ε4 carriers have increased levels of plasma inflammatory markers compared to noncarriers, and may also have a differential regulation of neuroinflammatory responses compared to other *APOE* isoforms.^46,47^ WMH might be a consequence of neuroinflammation.^48^

Our neuropathology data showed high agreement between our clinical diagnosis and the definitive pathologic diagnosis. Although our data showed that 100% of homozygous *APOE* ε4 carriers had CAA compared to 64% of heterozygotes, it did not show that WMH burden was associated with worse cognition in people with 2 alleles, and should be interpreted with caution due to the small sample size. While we cannot deduce that worse cognitive outcomes in *APOE* ε4 carriers with WMH are due to CAA, we can speculate that CAA is the more likely etiology for WMH in *APOE* ε4 carriers than in noncarriers, or the likelihood of CAA increases with each added *APOE* ε4 allele. The accelerated amyloid deposition in *APOE* ε4 carriers together with CAA may have a multiplicative detrimental effect on cognition. Findings from a recent population-based study concur with our data showing accelerated WMH-related decline in MMSE score in *APOE* ε4 carriers only. However, this study employed a microvascular lesion load summary score, which ranked an individual from 0 to 3 based on the absence or presence of WMH volume, lacunes, and perivascular spaces beyond a predefined cutoff. In addition, this study did not examine the effects of *APOE* ε4 allele dosage on the associations of microvascular lesion load and MMSE. Therefore, comparisons to our results in this regard could not be made.^49^ In contrast, we used quantitative WMH volume as a continuous predictor and 3 cognitive domains as outcomes rather than global cognitive score in our study.

We examine the effect of *APOE* ε4 on the association between WMH and cognition in the 2 most common neurodegenerative dementia diagnoses—AD and DLB—which is uncommon as most studies focus on AD. Strengths of our study include a well-characterized study sample of patients with dementia, rigorous image processing methods validated for older adults and mixed dementias, comprehensive neuropsychological testing, adjusting for confounders, use of an autopsy-confirmed subset of data, and replication of findings in an independent dataset. However, there are limitations. This was a cross-sectional study and therefore causal inferences could not be deduced. The statistical tests in some subanalyses, such as those in homozygous *APOE* ε4 carriers and the autopsy subsample, had limited power to detect associations, and the null association in the noncarriers of *APOE* ε4 might be a result of the limited sample size (power) as well. Therefore, studies with larger sample sizes are required. However, in an attempt to obtain more robust estimates, we conducted meta-analyses of estimates from SDS and ADNI, which resulted in stronger results. The SDS and ADNI-I used a different neuropsychological battery; however, there were similar tests available in both cohorts tapping into the major cognitive domains. This would not have affected our results as replication is more robust if performed using a different methodology to test the same research question. The number of patients who completed each cognitive test differed, which was related to dementia severity. Missing data from more severe cases might have resulted in an underestimation of the associations. Smoking and diabetes were not documented for most ADNI-I participants, hence were not included as covariates; these were not significant confounders in the SDS sample, so models in the 2 samples are fairly comparable. The numbers in the autopsy-based dataset were not sufficient to draw definitive conclusions; however, they provided important insights and can possibly direct future research.

*APOE* ε4 may influence the association of WMH with executive functions and language across the spectrum of AD and DLB. Our meta-analysis results showed significant associations of greater WMH volume with cognitive impairment across all 3 cognitive domains tested. Information about the *APOE* ε4 status of patients may be useful to understand the relative contributions of different pathologies to an individual's unique dementia syndrome, and to guide therapy as well. Future studies should aim to extend these findings to other dementia diagnoses and larger datasets. These findings emphasize the importance of WMH (as a marker of SVD) across the AD/DLB spectrum, and open avenues for further research to understand shared etiologies and risk factors across the dementias.

## Glossary

Aβ: β-amyloid
AD: Alzheimer disease
ADNI-I: Alzheimer's Disease Neuroimaging Initiative Phase I
ANOVA: analysis of variance
BDS: backward digit span
BNT: Boston Naming Test
CAA: cerebral amyloid angiopathy
CFA: Confirmatory Factor Analysis
CFI: comparative fit index
CI: confidence interval
CVLT: California Verbal Learning Test
DLB: dementia with Lewy bodies
DSST: Digit Symbol Substitution Task
FDS: forward digit span
MMSE: Mini-Mental State Examination
PD: proton density
PF-FAS: Controlled Oral Word Association task–Phonemic Fluency
RAVLT: Rey Auditory Verbal Learning Test
RMSEA: root mean square error of approximation
SDS: Sunnybrook Dementia Study
SF: Semantic Fluency
SRMR: standardized root mean square
SVD: small vessel disease
TIV: total intracranial volume
WCST: Wisconsin Card Sorting Test
WMH: white matter hyperintensities
WMS: Wechsler Memory Scale
